# Molecular mechanisms of proteinopathies across neurodegenerative disease: a review

**DOI:** 10.1186/s42466-019-0039-8

**Published:** 2019-09-16

**Authors:** Alexander P. Marsh

**Affiliations:** 1 School of Psychology, Cardiff University, Cardiff, UK; 2 School of Psychological Science, University of Bristol, Bristol, UK

**Keywords:** Neurodegeneration, Protein folding

## Abstract

**Background:**

Although there is a range of different symptoms across neurodegenerative diseases, they have been noted to have common pathogenic features. An archetypal feature shared between these diseases is protein misfolding; however, the mechanism behind the proteins abnormalities is still under investigation. There is an emerging hypothesis in the literature that the mechanisms that lead to protein misfolding may be shared across neurodegenerative processes, suggesting a common underlying pathology.

**Main body:**

This review discusses the literature to date of the shared features of protein misfolding, failures in proteostasis, and potential propagation pathways across the main neurodegenerative disorders.

**Conclusion:**

The current data suggests, despite overarching processes being shared, that the molecular events implicated in protein pathology are distinct across common neurodegenerative disorders.

## Background

Numerous neurodegenerative diseases (ND) share remarkably common pathogenic features in spite of the diversity of clinical symptoms [6, 7]. For many, their pathogenesis is linked by: the misfolding of proteins that aggregate within specific brain regions; significant neuroinflammation and increased oxidative stress of those areas; with final degeneration of neural tissue [7, 48]. However, the molecular mechanisms responsible for the process of this conformational change from proportionate, healthy, functional proteins to pathological, accumulated structures is not yet fully understood [7, 48]. There is new discussion in the literature that the mechanisms behind these pathogenic features of common NDs may be similar, linking these disease processes in a way that was previously thought distinct. This review will focus on the current evidence for the similarities between mechanisms of (1) protein folding and quality control; and (2) protein propagation, specifically with respect to the validity of a shared prion-like propagation hypothesis. In discussion, the inclusion of proteins will be largely limited to those only for which there is the clearest evidence base [49].

## Protein folding and quality control

In order for the approximately 15,000 proteins present in the neural proteome to fulfil their biological function, they often require folding consistent with exact instructions encoded in the amino-acid sequence [23, 44]. However, the number of potential conformations of even a small polypeptide (around 100 amino acids) is vast, around 1 × 10^18^ conformations [3]; furthermore their native states often have only marginal stability under normal physiological conditions [23]. It is unsurprising then, the process of protein folding and degradation needs to be well regulated to maintain cellular integrity and health, correcting errors that occur due to vulnerability conferred by the complexity of the process.

This vulnerability results in cells being faced with a continuous stream of misfolded and aggregated proteins, which require supportive ‘molecular chaperones’ to refold, degrade, and clear them to maintain proteome homeostasis [51]. Both protein misfolding and failures in chaperone demonstrate links between the pathogenesis of NDs.

### Protein Misfolding

Cellular aging, disease-related gene mutations, or proteotoxic stressors, like reactive oxygen species or toxins, can cause proteins to change conformation and become misfolded, escape cellular quality control and begin to aggregate as amorphous, oligomeric or fibrillar formations [8, 10, 51]. These aggregates have potential to overwhelm proteostasis, compromising cell function. Neurons are particularly vulnerable to damage by protein aggregation due to their polarisation and size, requiring unobstructed axonal transport to complete their function [47]. In addition, due to their post-mitotic nature, they are unable to dilute the misfolded proteins, associated waste products and subsequent toxicity, through cell division [10, 47].

Spires-Jones et al. [49] illustrates the pervasiveness of misfolded proteins across the primary NDs. Despite this apparent link across NDs, the common misfolded proteins (e.g. α-Synuclein, Aβ, Huntington etc.) are mostly distinct in terms of: biological function, location within the nervous system and native structural appearance [49]. However, in their pathogenic conformation many of these proteins share a β-sheet-rich tertiary structure that facilitates the formation of amorphous, oligomeric or fibrillar formations [8, 10, 51, 53]. When in this β-sheet-rich tertiary structure, there is some evidence these proteins interact with each other, causing conformational changes into non-native states [49, 50, 53]. With respect to this interaction, there is growing attention given to the overlap of misfolded proteins and their toxic effects on neurons across NDs.

Presence of α-synuclein in Lewy bodies throughout the cortex is a primary feature of Dementia with Lewy Bodies (DLB), however, α-synuclein has also been demonstrated in the dopaminergic neurons in a subset of Parkinson's disease (PD) patients and in the amygdala in c.60% of diagnosed Alzheimer’s disease (AD) patients [20, 49, 52]. Transactive response DNA binding protein 43 (TDP-43) inclusions are hallmarks of certain Frontotemporal Dementias (FTLD) and Amyotrophic Lateral Sclerosis (ALS; [41]) but have also now been demonstrated in DLB + AD, PD with or without dementia, and in around one third of mixed-dementia [37]. However, despite this cross-over, data has shown that proteins, such as α-synuclein, can lead to specific ND phenotypes (such as those of FLTD, PD and ALS) without involvement of other misfolded proteins [49]. Given the presence of cross-over between proteins and studies demonstrating their interaction in non-native conformation [19, 49], it is conceivable to speculate they share a linked function in the pathogenesis of NDs. However, our current understanding of multimorbidity within NDs is limited and the current literature describes considerable heterogeneity between cases, making it difficult to clarify their contributions and interactions [49]. Additional large-scale quantitative analyses of post-mortem tissue from ND patients alongside clinical phenotypic data would aid clarification in the significance of these protein cross-overs, interactions and their linked contributions across NDs [12, 49].

The presence of misfolded proteins and their aggregation may be caused by heritable gene mutations in disease proteins, for example, in Huntington’s disease (HD) and in early onset AD and PD, and many ND cases demonstrate stochastic genetic mutations [23]. However, in the first study of its kind, a comparison of genome-wide gene expression data of 93 brain tissue samples from patients with AD, HD, Multiple Sclerosis, AML and PD demonstrated that, despite significantly high number of dysregulated genes in individual diseases, hardly any single specific genes demonstrated commonality between the NDs; those that did were primarily implicated in the innate immune system and neuroinflammation [12]. Given this finding, it appears at the genomic level there is not a single shared mechanism across NDs. It is however, important to note there are significant methodological issues with such a large comparative study and the methods used to standardise for analysis may have yielded an underrepresentation of shared genetic features [12]. Additionally, genome data in the absence of assessment of protein expression and post-translational modifications may be misleading and therefore proteomics should be explored in future work in order to rule out a potential shared pathogenic mechanism.

There has been further debate in the shared contribution of different protein aggregate species and their cytotoxic effects in NDs. Numerous studies have posited that oligomers pose greater toxic threat than fibrillary aggregates for amyloid proteins, including β-amyloid and TDP-43 [19, 53], though this appears to be inconsistent for aggregates of α-synuclein and huntington, in which fibrils have been demonstrated to be highly toxic [43, 56].

Overall, it appears that protein misfolding is a key player in the pathogenesis of many NDs, though the specific proteins implicated, their aggregates and the genetic basis of their pathology do not appear to be shared across all NDs. Further evidence is required to clarify the co-existence of proteins and their interactions in co-morbid NDs.

### Chaperones

**Fig. 1:**
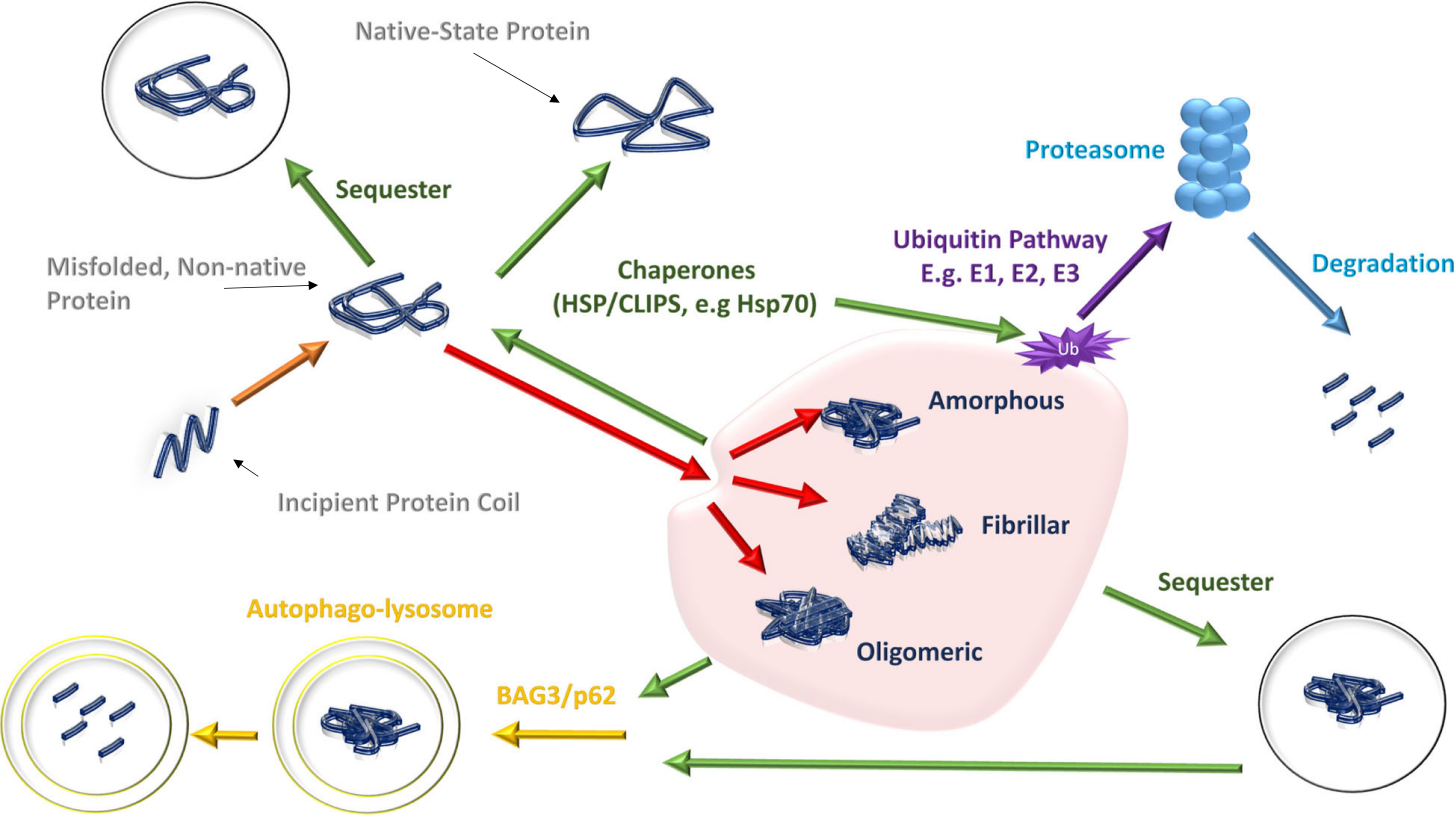
Quality Control of Misfolded Proteins. Green arrows denote chaperone pathways. When a misfolded protein occurs, it can be detected by a molecular chaperone and refolded into a native protein. If this process cannot be completed, either because the native protein is unable to undergo further conformational change or because it has formed an aggregate, it can be sequestered and degraded at a later stage. When protein aggregates form in either an amorphous, fibrillary or oligomeric state, chaperone proteins can initiate two destruction responses. They can target them for destruction via the Ubiquitin-proteasome pathways by facilitating ubiquitin tagging, or facilitate action via BAG3/p62 them for autophagal degradation. ER = Endoplasmic Reticulum; E1 = ubiquitin–activating enzymes; E2 = ubiquitin–conjugating enzymes and E3 = ubiquitin ligases. [Based on Reference Hartl [23] and Tofaris & Buckley [52]]

Chaperones bind to incipient proteins when they leave the ribosome as random coils and support in their folding into 3D-structures. In addition, they quality check the proteins are correctly folded and either redirect non-native species to their native state or target them for (1) degradation through the ubiquitin proteasome system; (2) degradation via the autophagy pathway; or (3) sequester them into transient or stable deposits within cellular compartments when degradation fails (see Fig. 1; [23, 51]). Functional and genomic analyses have demonstrated two distinct subsets of chaperones: Chaperones Linked to Protein Synthesis (CLIPS), which are supressed by stress, primarily involved in protein folding and are transcriptionally co-regulated with translational apparatus; and Heat Shock Proteins (HSPs) that are stress induced and are primarily involved in prevention of protein aggregation [1].

Several studies have directly implicated human mutations in Valosin-containing protein(VCP), Hsp70 and Hsp40 chaperone genes in FTLD, PD and ALS [28, 54, 57]. Brehme et al. [8] found particular subsets of chaperones, primarily CLIPS, exhibit repressed transcription during ageing (the most significant and conserved risk factor across NDs), and that this repression is greater in brains of AD, HD and PD patients, further implicates a link between chaperone expression and the risk for and pathogenesis of multiple NDs. Brehme et al. [8] further discovered, in knockdown models of *C. elegans*, the genes for these chaperones were essential in the prevention of proteotoxicity during normal ageing and exacerbated phenotypes of induced AD and HD via increased protein aggregation. In further support, Eroglu et al. [15] demonstrated knockout of a well-conserved chaperone (Hsp110) in mice led to accumulation of hyperphosphorylated-tau and subsequent neurodegeneration; furthermore, in mice overexpressing APP, Hsp110 knockout led to appearance of insoluble amyloid β42.

Wacker et al. demonstrated Hsp70 loss led to significantly increased size of inclusion bodies formed by mutant huntington (Htt) and had subsequent exacerbation of the physical and behavioural biomarkers of HD. Interestingly, deletion of the Hsp70 chaperones affected inclusion bodies but, paradoxically, did not impact the levels of fibrillary aggregates caused by Htt. The authors suggest Hsp70s likely target inclusion bodies that are not on path to form fibrils but nonetheless still pose deleterious effects on the cell and contribute to cell death. Other evidence from immunofluorescence studies supports this hypothesis, suggesting that Hsp70s serve a role in sequestering inclusion bodies, whilst a different chaperon, Hsp90, serves a crucial role in prevention of fibril aggregation [22]. These findings speak to the importance in preservation of different chaperones and how their selective dysfunction leads to multiple pathologies in ND.

Multiple studies have demonstrated that chaperones promote removal of pathogenic misfolded proteins and their aggregate forms in many NDs [10]. McLear et al. demonstrated that enhanced expression of Hsp70 in established drosophila models of HD dramatically improved survival and lifespan, though did not demonstrate complete resolution of the HD phenotype [36]. This supports earlier discussion of the specific contribution to pathogenesis of Hsp70 mutations. Labbadia et al. [32] demonstrated similar findings in mammals, showing that overexpression of HSJ1a (a co-chaperone to Hsp70) significantly reduced Htt-aggregates and subsequently improved behavioural performance on a variety of activity and motor assessments in R6/2 mouse models of HD. McLean et al. used immunocytochemistry to demonstrate that Hsp70 co-localised with 66% of lewy bodies found in human cell culture of PD and AD patients; furthermore they demonstrated that over-expression of Hsp chaperones prevented α-synuclein aggregation in a human-cell model [35]. Evans et al. added to this work, demonstrating that recombinant Hsp70/40 and Hsp90 block Aβ self-assembly in in vitro human-cell culture. Consistent with evidence in other ND models [22, 35, 56], they found that these chaperones supressed early stages of self-assembly, altered pre-formed oligomers but had little effect on fibrils. Evidence in familial-ALS transgenic mice models failed to show any benefit of upregulated Hsp70 [33], however, overexpression of HSJ1a did demonstrate significant improvement in muscle force, increased motor unit number and enhanced motor neuron survival as well as reduced SOD1 aggregation [42].

Overall, these data, alongside the studies of chaperone failures, demonstrate a convincing link between NDs through the contribution of chaperones to pathogenesis and as a shared potential treatment target. However, they also demonstrate ‘not one size fit all’ phenomena, as they implicate differing contributions of these molecular units to different ND types, such as the greater impact of HSJ1a in the motor NDs discussed compared to Hsp70 in AD.

## Protein Propogation

The focus of this section will be on the favoured, emerging hypothesis for protein propagation; which is the proposition of a non-cell-autonomous process, in which non-native protein species propagate in a prion-like manner from a ‘donor cell’ to an ‘acceptor cell’ and proliferate by recruiting native proteins and transforming them into proteotoxic conformers [29, 51, 53, 55]. There is significant debate whether the proteins in NDs are true prions, as in Prusiner’s original paper [45], and to what extent they share common features [53]. Despite the discussed differences in prevalence, initial structure, function and location of the proteins implicated in NDs, it could be considered they utilise common pathways for their propagation in a prion-like way by misfolding native proteins. However, unlike true prions, ND proteins are not strictly speaking ‘infectious’, in that they are unable to exit the body, travel to another organism and resume replication under natural conditions [29, 53]. In two large studies, no infective transmission between humans of AD and PD occurred in cadaver-derived human growth hormone (HGH) recipients [25] or blood transfusion recipients [4] and to date there are no reports of induced-ND following organ transplant, as would be expected in typical prion diseases, such as Creutzfeldt-Jakob disease [4, 50]. However, one study did report that half of the eight cases examined demonstrated potential transmission of Aβ-plaques via pituitary-HGH transplant in patients demonstrating AD pathology post-mortem, with low risk factors of disease development [26]. Further epidemiological data of patients receiving biomaterial from ND-patients would help clarify these inconsistent findings and the true infectious nature of ND protein aggregates.

Notwithstanding, many studies demonstrate support of a prion-like model of spread within NDs [19, 29, 50, 53, 55]. The earliest evidence from Goudsmit et al. [18] failed to support the model; non-human primates were inoculated intracerebrally with brain tissue from 52 patients with AD, following which they developed an encephalopathy consistent with CJD but failed to reproduce AD. However, it is important to note the incubation time prior to examination was brief in respect to AD progression. In a similar study, three non-human primates were intracerebrally inoculated and sacrificed 6–7 years after. Histology of their brain tissue was compared with colony aged-matched controls and revealed significant presence of Aβ-plaques consistent with AD pathology but no neurofibrillary tangles. Subsequent work with transgenic mice supported this finding. β-amyloid-containing brain extracts from two strains of aged transgenic AD mice models were transplanted into the hippocampus of young mice. Following transplantation, the young mice developed AD pathology consistent in terms of morphological, conformational, and Aβ40:Aβ42 ratio characteristics with the strain transplanted [24], which is consistent with other evidence of amyloid-β acting in a prion-like manner [13, 14, 50].

Tau fibrils have demonstrated the ability to enter ‘acceptor cells’ and cause fibrillisation of native-tau in cell culture studies [50, 58]. When brain extracts from humans that died from tauopathies are injected into the hippocampus of mice transgenic for wild-type human tau, argyrophilic tau inclusions form and recapitulate the ND phenotypes [11]. Kaufman et al. was able to use two transgenic tau strains to cause strain-specific pathology in distinct cell types and brain regions as well as provoke strain-specific rates of network propagation [30].

Two case studies demonstrated that pathological changes, including Lewy body–like structures that stained for α-synuclein, can develop in human foetal neurons grafted into a host with PD disease [31], demonstrating prion-like transmission from host to graft. Later animal work supported this finding, demonstrating in vivo transfer and interaction of α-synuclein between host cells and grafted dopaminergic neurons in mice overexpressing human α-synuclein [21]. Further evidence demonstrated injected α-synuclein fibrils recruit host-cell α-synuclein into pathological aggregates that spread transneuronally over 1–12 months after injection [46].

Drosophila models of HD have demonstrated interneuronal spreading of Htt aggregation in a prion-like manner [2]. Cicchetti et al. [9] described the presence of Htt derived oligomers within grafted striatal tissue in three HD patients from c.10 years prior, whom later died secondary to the progression of HD. Further studies in both culture and in vivo animal work have demonstrated prion-like propagation of Htt [27, 50].

ALS and FLTD-related proteins, including SOD1 and TDP-43, have been shown both in stem-cell culture and animal studies to follow self-perpetuating seeded aggregation, consistent with prion-like transmission [34, 50, 59].

These data outline evidence of prion-like propagation in several major NDs, however, it is still not clear quite how prion’s escape the macropinosome and transmit between cells [50]. Converging evidence does support hypotheses of utilisation of lysosome and tunnelling nanotubules [53], though the mechanisms by which tunnelling nanotubules are formed and how protein aggregates recruit them is still unclear [53]. In addition, mechanisms including membrane disruption, release via exosomes, secretion of soluble material, and cell death have also been proposed though the evidence is inconsistent and requires further study [50, 59].

If the prion hypothesis were to hold true, therapeutic attempts to enhance extracellular clearance of misfolded proteins, inhibition cellular uptake and intracellular aggregate seeding, and disrupt aggregate release into extracellular space would be effective approaches [50]. However, to date such attempts have been inconsistent. Immunotherapeutic approaches promoting extracellular clearance of misfolded proteins in AD and synucleinopathies have had some moderate success in animal models [5, 60] and there are now several clinical trials in AD and PD [50]. However, success has been inconsistent with multiple phase III clinical trials failing [17, 60, 61]. One potential reason for this translational failure, however, is that the animal model studies had a priori knowledge of disease presence and consequently were able to intervene earlier in disease progression than possible in the clinical trials, were there was a minimum of presence of modest cognitive impairment. Later phase III trials, have done well in improving on this and are now recruiting at-risk and asymptomatic patients, more consistent with the animal study protocols [38–40]. Treatments targeting protein aggregation also have demonstrated poor clinical efficacy in clinical trials [16], though again these targeted mild-to-moderate disease states and may be administered too late to have therapeutic impact.

Overall, despite significant and promising evidence across NDs for protein propagation in a prion-like manner, until we can better characterise the intracellular protein transmission mechanisms and demonstrate that specific interventions are able to block this prion-like behaviour, a prion model of ND propagation cannot be confirmed.

## Concluding remarks

It is clear that NDs are not entirely distinct diseases and they share many common themes, including protein misfolding, failures in proteostasis, and potentially propagation pathways (though this is yet to be confirmed). However, despite these overarching, similar features, the molecular components implicated, such as the specific proteins and molecular chaperones involved, and the risk factors that are associated with those components, do not appear to be shared across all NDs and speak to the pathological specificity of the molecular mechanisms behind different NDs.

## Data Availability

Data sharing not applicable to this article as no datasets were generated or analysed during the current study.

## Abbreviations

AD: Alzheimer’s disease
ALS: Amyotrophic Lateral Sclerosis
CLIPS: Chaperones linked to protein synthesis
DLB: Dementia with lewy bodies
FTLD: Frontotemporal Dementias
HD: Huntington’s disease
HSP: Heat specific protein
Htt: Mutant Huntington protein
ND: Neurodegenerative disease
PD: Parkinson’s disease
TDP-43: Transactive response DNA binding protein 43
